# Child–Computer Interaction at the Beginner Stage of Music Learning: Effects of *Reflexive Interaction* on Children’s Musical Improvisation

**DOI:** 10.3389/fpsyg.2017.00065

**Published:** 2017-01-26

**Authors:** Anna Rita Addessi, Filomena Anelli, Diber Benghi, Anders Friberg

**Affiliations:** ^1^Department of Education Studies, University of BolognaBologna, Italy; ^2^Department of Speech, Music and Hearing, KTH Royal Institute of TechnologyStockholm, Sweden

**Keywords:** reflexive interaction, children’s music improvisation, child–computer interaction, assessment of children’s performance, MIROR-Impro

## Abstract

In this article children’s musical improvisation is investigated through the “reflexive interaction” paradigm. We used a particular system, the MIROR-Impro, implemented in the framework of the MIROR project (EC-FP7), which is able to reply to the child playing a keyboard by a “reflexive” output, mirroring (with repetitions and variations) her/his inputs. The study was conducted in a public primary school, with 47 children, aged 6–7. The experimental design used the convergence procedure, based on three sample groups allowing us to verify if the reflexive interaction using the MIROR-Impro is *necessary* and/or *sufficient* to improve the children’s abilities to improvise. The following conditions were used as independent variables: to play only the keyboard, the keyboard with the MIROR-Impro but with not-reflexive reply, the keyboard with the MIROR-Impro with reflexive reply. As dependent variables we estimated the children’s ability to improvise in solos, and in duets. Each child carried out a training program consisting of 5 weekly individual 12 min sessions. The control group played the complete package of independent variables; Experimental Group 1 played the keyboard and the keyboard with the MIROR-Impro with not-reflexive reply; Experimental Group 2 played only the keyboard with the reflexive system. One week after, the children were asked to improvise a musical piece on the keyboard alone (Solo task), and in pairs with a friend (Duet task). Three independent judges assessed the Solo and the Duet tasks by means of a grid based on the TAI-Test for Ability to Improvise rating scale. The EG2, which trained only with the reflexive system, reached the highest average results and the difference with EG1, which did not used the reflexive system, is statistically significant when the children improvise in a duet. The results indicate that in the sample of participants the reflexive interaction alone could be sufficient to increase the improvisational skills, and necessary when they improvise in duets. However, these results are in general not statistically significant. The correlation between Reflexive Interaction and the ability to improvise is statistically significant. The results are discussed on the light of the recent literature in neuroscience and music education.

## Introduction

In his book “The improvising mind. Cognition and creativity in the musical moment,” Berkowitz (2010), explores the field of ability to improvise through an interdisciplinary approach (i.e., musical analysis, neuroscience, historical pedagogical methods on improvisation, interviews with musicians), showing the complexity of the phenomenon of improvisation and, at the same time, the complexity of studying music improvisation. In the field of music education, several scholars approached the problem from different perspectives and by means of different methodologies, giving rise to discussion about the definition of improvisation in children’s musical experience, the relationships between improvisation and creativity, learning/teaching theories and social/familial contexts. Baroni (1997) describes several examples of children’s improvisation in the context of expressive activities in a classroom setting (kindergarten and primary school); Brophy (2005) carried out a longitudinal study of selected characteristics of children’s melodic improvisation; Delalande (1993) used the concept of “conduct” to observe the musical improvisation of children on the basis of the Piagetian concepts of sensory-motor, symbolic and rules games; Hickey and Lipscomb (2006) also offer a model of assessment of children’s creative musical thinking; Kratus (1996) established several levels of improvisation: exploration, process-oriented improvisation with the presence of some micro-structures, product-orientated with four more levels of relationship between micro and macro-structures; McPherson (1993, 2005) elaborates a grid to assess the student’s ability to improvise; Mialaret (1997) proposes a model to classify children’s instrumental musical explorations; Tafuri (2006) describes several models of musical improvisation with children; Young (2004), introduces the interpersonal perspective on childrens’ music improvisation together with the teacher; Webster (2002) studies children as creative thinkers in music and proposes a model of creative thinking in music.

In our study, we investigated this issue through the “reflexive interaction paradigm.” This paradigm describes a particular kind of human–machine interaction where the users can interact with a virtual copies of themselves, through specific software called interactive reflexive musical systems (IRMS). The first prototype of IRMS, the Continuator (Pachet, 2003) was originally conceived for adult musicians. However, we decided to experiment it with children and the exploratory study (e.g., Addessi and Pachet, 2005, 2006) immediately showed that these systems can have a strong impact on the development of children’s creative musical experiences, introducing a new perspective in the field of technology-enhanced learning In this field, most studies deal with internet devices, teaching strategies, composition, performance, and music therapy: the new technology opens new scenarios on musical learning and teaching (e.g., Delalande, 2003; Brown, 2007; Webster, 2007; Williams and Webster, 2008; Finney and Burnard, 2009; Dorfman, 2013; Bauer, 2014). However, new technology can be considered not only as a “tool” to aid teaching, but also as providing languages and “brainframes” (Turkle, 1984; De Kerckhove, 1991) that deeply influence the processes of musical learning and the musical creativity of children. Recently, several studies have been realized to analyze human–machine interaction and the evaluation and design of interfaces for collaborative music making, focused on user experience, and affective and emotional behavior: these studies were mainly focused on adults and adolescents (cfr. Morgan et al., 2014).

**FIGURE 1:**
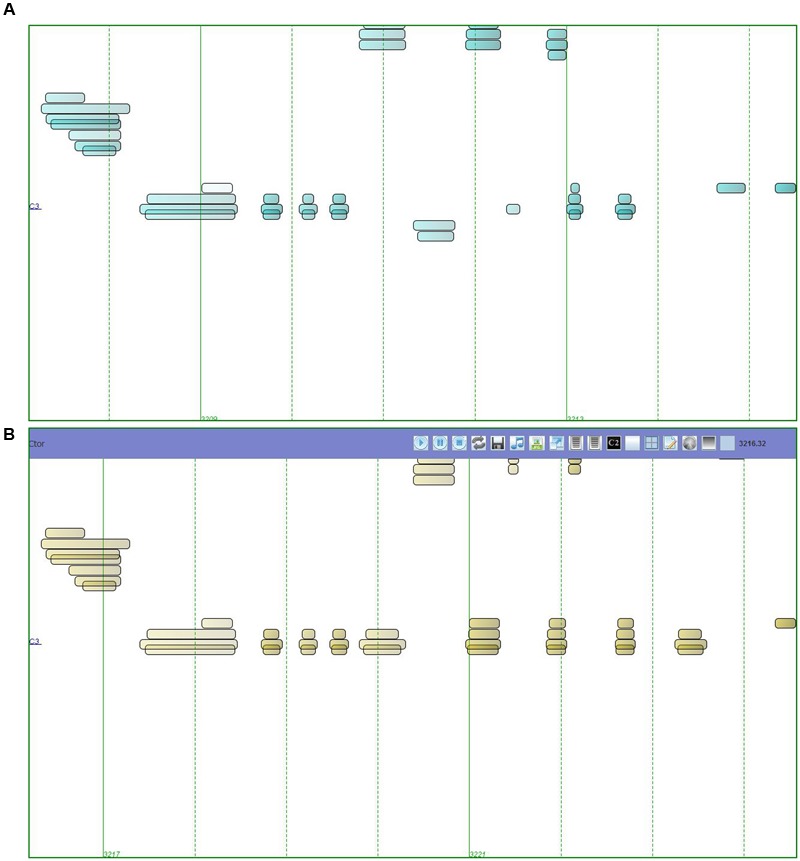
**Piano roll representation: A musical input played by a child (A)** is continued by the MIROR-Impro **(B)**, with repetition and variation.

Our study aims to investigate whether the reflexive interaction using the IRMS influences the children’s skillfulness to improvise, at the beginner stage of musical learning. We used a particular IRMS, the MIROR-Impro, implemented in the MIROR project (EC-FP7), which is able to reply to the child playing a keyboard by mirroring (with repetitions and variations) her/his inputs (Pachet et al., 2011; Addessi et al., 2013). During a session with the MIROR-Impro, the user plays a (Midi) keyboard and, when she/he stops, the system immediately answers with a musical phrase similar to the input played by the user. A dialog then takes place between the user and the machine, in which the two partners repeat and vary the musical ideas. The user’s inputs are analyzed by the MIROR-Impro to gradually build a model of the user. **Figures 1A,B** show how a simple musical input played by a child (**Figure 1A**) is continued by the MIROR-Impro (**Figure 1B**) with repetition and variation.

### The Interactive Reflexive Musical Systems

Pachet (2003, 2004, 2006) considers reflexive interactive systems as a particular “class of interactive systems in which users can interact with virtual copies of themselves, or at least with agents that have a mimetic capacity and can evolve in an organic fashion” (2006, 360). He identified several features that characterized reflexivity but were to be considered “by no means exhaustive” (2006, p. 360):

• *Similarity or mirroring effect:* The IRMS “produces musical sounds similar to what the user is (…) able to produce. This similarity must be easily recognizable by the user, who must experience the sensation of interacting with a copy of her/himself” (2006, p. 360).
• *Agnosticism:* “The system’s ability to reproduce the user’s personality is learned automatically and agnostically, -i.e., without human intervention.” In the case of the Continuator, “for instance, no pre-programmed musical information is given to the system” (2006, p. 360).
• *Scaffolding of complexity.* “Incremental learning ensures that the (IRMS) keeps evolving and consequently that the user will interact with it for a long time…. Incremental learning is a way to endow the system with an organic feel, typical of open, natural systems (as opposed to pre-programmed, closed-world systems)” (2006, p. 361).
• The turn-taking between the user and the system is the *basic playing mode* of the IRMS. It is organized on the basis of three principles: (1) “Automatic detection of phrase endings”; (2) Setting the duration of the phrase generated by the IRMS “to be the same as the duration of the last input phrase”; (3) Giving “priority to the user” (Pachet, 2004).

The principle of “similarity and mirroring” is particularly important for the definition of the IRMS: “the musical output must typically lie in between two extreme forms of musical production: Repetition and Randomness. Repetition is obtained by echoing musical elements of the user, without any reorganization. Repetition creates a sense of mirroring, but does not exhibit any increase in complexity. Randomness can exhibit complexity but is not related to the user’s personality (2006, p. 362)”. The system’s output is generated by means of the Markov probabilities calculated during the analysis of the musical input. In the framework of the MIROR project, Pachet et al. (2011) recently proposed using the Markov probabilities to generate musical sequences satisfying several constraints, such as the starting and the ending notes. According to this perspective, the system output does not imitate exactly what the musician is doing, but rather imitates her/his musical style. The notion of style of IRMS consists “of the statistical distribution of notes, chords and musical elements in general as well as their ordering” (Pachet, 2004, p. 3).

### Observations and Theoretical Framework of Reflexive Interaction

In our previous work, we tried to explain the human behaviors during the interaction with a reflexive system, starting from the observation of the interaction between children and the IRMS (Addessi, 2014). Our aim was to examine the human reflexive behavior, both in human–machine and human–human interaction, to understand how reflexive interaction can support and enhance creativity and the learning/teaching processes. We suggested that the idea of mirroring originated in ancient Western culture and now resonates with contemporary theory of musical embodiments and the mirror system.

An example of micro-analysis featuring this mechanism and how it develops is provided by the interaction between the MIROR-Impro and a 7-year-old girl, video-recorded during the experiment introduced in this article:

*The little girl plays two consecutive notes, C2 and A2, and then stops to wait for the response of the system. The system responds by repeating the same notes. The child then plays a single note, G2, and the system responds with a single note but this time introduces a variation: she plays C3, thus introducing a higher register. The girl, following the change introduced by the system, moves toward the higher register and plays a variant of the initial pattern, namely: D2-A2-E2-C3, and introduces a particular rhythm pattern. This “reflexive” event marks the beginning of a dialog based on repetition and variation: the rhythmic-melodic pattern will be repeated and varied by both the system and the child in consecutive exchanges, until acquiring the form of a complete musical phrase. At some point in the dialog, the child begins to accompany the system’s response with arm movements synchronized with the rhythmic-melodic patterns, creating a kind of music-motor composition*.

In this example, as well as several examples reported in our previous works (e.g., Addessi and Pachet, 2005; Addessi, 2014), it is possible to observe how:

• The attention of the young girl increases when the system imitates her musical sentences and decreases when the system’s output become more varied;
• The interaction between the child and the machine is not predetermined by the machine, nor realized only by the child, but is co-constructed by the child along with the machine;
• The *co-regulation* (Fogel, 2000) is based on a continuous *repetition* and *variation* mechanism between input and output data from the child and the system;
• The partners are able to imitate each other and the child recognizes being imitated;
• The interaction is based on *turn-taking*: the child plays, then stops, waiting for the response of the system and when it comes she listens to it carefully, perceives its reflexive qualities and in turn the child responds by imitating and varying the system’s response;
• The response of the system takes up the last input played by the child, giving rise to a *regular timing of turns*.
• The child dialogs with the system by means of sounds, which is an evident manifestation of *thinking in sound* (McPherson, 2005).

What it is interesting in this kind of child–machine interaction, is the creation of a dialog between the child and the system, which shows some *biological constraints* as described in Katayose (2014): physical fatigue, sensory error, and, above all, balancing between repetition and novelty. The repetition and variation mechanism is, in fact, the fundamental characteristic of reflexive interaction: the girl in the microanalysis starts to show an absolute attraction toward the system’s reply precisely when she recognizes the system’s repetition of her input. It is interesting to note that it is not merely a repetition, but rather a repetition that is constantly varied. The co-presence of repetition along with something different seems to attract and stimulate the user to become involved in the interaction. The topics of *mirror* and *sound mirror* are very ancient in Western culture: from the myth of Echo and Narcissus (Ovid, 43 B.C.-18, *Metamorphoseon libri XV*), to the antiphonal *echo* effects of renaissance and baroque music, to the power of music to reflect the human affects of the *Teoria degli affetti* and the *Affektenlehre* (Galilei, 1581; Kircher, 1650), More recently, the mechanism of repetition and variation is at the heart of the semiological paradigmatic analysis (Ruwet, 1966) and the theory of similarity perception in listening to music (Deliège, 2001; Toiviainen, 2007). The imitation of the behavior of others seems to be grounded on the non-conscious processing known as the *chameleon effect* (Lakin and Chartrand, 2003). Studies in neuroscience root these non-conscious mechanisms in the mirror neuron system (MNS), a network of neurons that becomes active during the execution and observation of actions, on the basis of a “resonance mechanism” (Rizzolatti et al., 2002). Recent studies in psychology and neurosciences increasingly suggest that the mechanisms of repetition and variation, imitation, recognition, and self-imitation, play an important role in infant musicality development and in the ontological fundamentals of human musicality (e.g., Bruner, 1983; Dissanayake, 2000; Meltzoff and Prinz, 2002; Stern, 2004; Imberty, 2005; Papousěk, 2007; Gratier and Apter-Danon, 2009; Malloch and Trevarthen, 2009). The majority of this research refers to the ability of children to imitate, described by Meltzoff as the *like-me* mechanism: “persons are like-me entities in so far as they can do like me, and I can do like them” (Nadel, 2002, p. 46). Anzieu (1996) introduced the concept of *musical wrapping* of the Self, to describe the original infant-mother relationship, characterized by the presence of the most archaic forms of repetition: the *echo*. For this raison, reflexive interaction can also be described as a “*dynamic process*” (Addessi, 2014, p. 219): the experience of repetition and variation that the infant experiences during the vocal interaction with the adult in the 1st months of life, is realized in the framework of affective and emotional valence, that is the *amodal* experience that Stern (2004) calls *affective contours*. The fundamental role of the body in human musical activities is underlined also by the recent studies carried out in the field of embodied music cognition, which can explain the relationship between reflexive interaction and body perception. In particular way, it is important to underline the nearness of the reflexive interaction paradigm with the acoustic metaphor of “resonance mechanism,” used by Rizzolatti et al. (2002, p. 253) to describe the correspondence between “observed and executed biological motion” in the framework of the mirror neurons system. Leman (2007, p. 91), underlines that “mirror neurons are amodal, in the sense that they can encode the mirroring of multiple sensory channels.” Therefore, “reflexive interaction would stimulate a resonance mechanism in the child interacting with an IRMS. This resonance could have a neural basis in the MNS” (Addessi, 2014, p. 220). At the same time, interactive learning and the development of individual musical expressivity has been investigated in various studies that highlight the complex multi-modal character of these processes. Prinz (2008) described a *common coding* theory, which posits a shared representational domain for perception and action among individuals based on a mirroring mechanism, which can explain the neurobiological fundamental of reflexive interaction.

### Is Reflexive Interaction Necessary and/or Sufficient to Improve Children’s Ability to Improvise?

The basic hypothesis of our study is that reflexive interaction (in short RI) can “enhance music learning and musical creativity in young children (…). The pedagogical potential of reflexive interaction is based on the fact that it stimulates the subject to undertake a dialog during which the repetitions and variations enhance cognitive conflict that the child resolves during the course of the interaction, giving rise to a learning by problem finding and problem solving” (Addessi, 2014, p. 223). It was observed that the reflexive interaction using IRMS stimulated and reinforced children’s exploratory conducts, characterized by the use of variations, in a variety of elements: melody, rhythms, gestures, registers, speed, dynamics, phrasing. The discovery that the system replies by imitating them, motivates the children to undertake a musical dialog with the system, which is the basis for the elaboration of particular sounds and musical ideas. It was possible to observe that each child expressed her/his individual musical style, in producing the sounds, handling the keyboard and the other equipment, and her/his style was supported and reinforced by the mirroring effect of the system’s output. It is possible to affirm that, during the reflexive interaction, the musical invention is a collaborative experience of playing: the child/ren and the system, in a pair, improvise together like two musicians. It was observed that reflexive interaction using the IRMS increases the attention span, stimulates attentive listening, intrinsic motivation, musical creativity, and ability in collaborative improvisation (e.g., Addessi and Pachet, 2005, 2006; Addessi et al., 2013; Ferrari and Addessi, 2014; Rowe et al., 2015). The dialog with a “virtual” musician stimulates children to “think in sound,” which is considered one of the main aims of music education (McPherson, 2005). IRMS establish an interaction between pairs, which exploits the Vygotskian concept of zone of proximal development (ZPD) (Vygotsky, 1962). The mirroring mechanism creates a balance between challenges and skills, which enables the MIROR application to create children’s flow experiences and creative processes (Csikzsentmihalyi, 1990; Pachet, 2004; Addessi et al., 2015).

**FIGURE 2:**
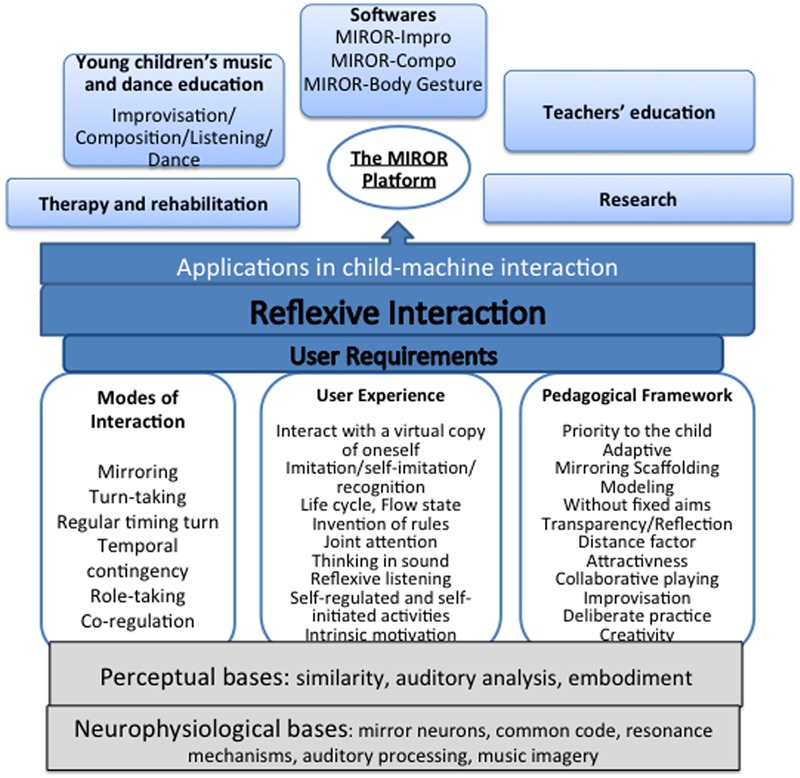
**Theoretical framework of reflexive interaction with implication for child–computer interaction (as from Addessi, 2014)**.

The pedagogical potential of IRMS was fully exploited in the framework of the MIROR project, to enhance children’s collaborative playing, self-regulation, self-initiated activities, and a learner-centered approach. These pedagogical strategies are already used in some educational contexts, including music education (e.g., Custodero, 2005). But we underline that in the case of the MIROR applications, these strategies and behaviors occur between the children and the machine, a mechanism that is not very usual in the field of child–machine interaction and in the field of technology-enhanced learning. **Figure 2** shows the theoretical framework of reflexive interaction with implications for child–computer interaction.

The questions that lie at the basis of our study are as follows: does the reflexive interaction by means of the MIROR-Impro affect children’s ability to improvise? In particular, does it affect the ability to improvise a musical dialog with a partner? Can the MIROR-Impro be used as a teaching device at the beginner stage of learning musical improvisation? In our pilot studies and investigations on children and IRMS, we observed several features of child–machine interaction, based on reflexive interaction, which led the children to improvise musical duets with the system (cfr. Addessi and Pachet, 2005). These “musical conversations” were based on the mechanism of repetition-variation, which is “at the heart of reflexive interaction” (Addessi, 2014, p. 2017). We observed and measured the flow emotional state (Csikzsentmihalyi, 1990) of children playing with the MIROR-Impro (Addessi et al., 2015). We showed that the flow experience increases when children play with the system and, in particular, when the system’s reply is more reflexive. The experience of flow is often used to describe the sense of complete absorption in the act of improvising, in which the improviser seems to merge with the music and transcend everyday consciousness (Berkowitz, 2010, p. 127) and has been used as a tool to investigate human–machine interaction (Leman et al., 2010; Nijs et al., 2012). Therefore, we could argue that reflexive interaction is able to enhance the experience of musical creativity and improvisation. Further studies conducted in the framework of the MIROR project highlight the children’s perspective on their own improvisation (e.g., Triantafyllaki et al., 2012; Cardoso de Araújo and Addessi, 2013; Rowe et al., 2015) and some musical patterns evident in their improvisations with the MIROR-Impro (Alexakis et al., 2013). Nevertheless, there are still no studies that show in a controlled experimental design that IRMS positively influence in a particular way the ability to improvise.

The aim of the present study is to investigate whether the use of reflexive interaction with the MIROR-Impro is “necessary” and/or “sufficient” to improve children’s abilities to improvise, and, consequently, whether it could be useful to include the MIROR-Impro in a program of teaching improvisation. Moreover, in our study, we studied two specific conditions of improvisation: solo and duet performance.

## Materials and Methods

### Participants

The study sample consisted of 47 children, aged 6–7, distributed in two natural classroom groups in the 1st year of a primary school. Participants were randomly divided into three sample groups: 16 children for the Control group (CG), 15 children for the Experimental Group 1, and 16 children for the Experimental Group 2. The children had not attended any formal course of instrumental education. They were used to carrying out general classroom activities in music education with the teachers.

### Equipment

The software MIROR-Impro, a component of the MIROR Platform (v.3.14). Three different setups of the system’s output were used: setup Nothing, the system did not reply; setup Similar, the system’s outputs were similar to the input of the child; setup Very Different, the system’s outputs were totally different from the child’s input. As can be seen, only the setup Similar used reflexive interaction. In addition: music synthesizers KORG X50; notebooks; headphones; amplifiers M-AUDIO AV30; USB cables for the connection between the synthesizer and the notebook; video cameras (recording in HD).

### Experimental Design

The convergence procedure (Campbell and Heller, 1979; Fiske, 1992) was used, based on three sample groups (one control group - CG - and two experimental groups -EG1 and EG2), allowing us to verify not only if the reflexive interaction using the MIROR-Impro affects the children’s musical improvisation but also, more precisely, if it is “necessary” and/or “sufficient” to improve the children’s abilities to improvise, in solos and in duets. This procedure is based on the comparison of three different experimental conditions, represented by three groups of participants: CG, Experimental Group number 1, and Experimental Group number 2. Each experimental condition represents a possible combination of different independent variables, one of which is reflexive interaction.

In our protocol the following three independent variables were considered:

(1) Playing the keyboard (v1): in this case, we used the MIROR-Impro system with the setup Nothing and, as a consequence, the system did not produce any output;
(2) Playing the keyboard with the MIROR-Impro but with not-reflexive reply (v2). In this case we used the MIROR-Impro system with the setup Very Different: in this setup the system’s reply is very different from the input played by the children and there is no reflexive interaction. For example: the child plays a long cluster, loud, with the palms of the hands in the higher register, and the system replies with three short single notes in the low register.
(3) Playing the keyboard with reflexive interaction using the MIROR-Impro (v3): in this case we used the setup Similar in which the system repeats the child’s input with variations and a reflexive interaction is thus started between child and system. For example: the child plays a long cluster, loud, with the palms of the hands, and the system replies with a short cluster in the same register followed by an ascending *arpeggio.*

The convergence procedure focuses the attention on one of these variables, which is manipulated in order to determine if the variable is *necessary* to enhance the dependent variables, and *sufficient* to enhance the dependent variables. In our protocol the independent variable to be controlled is variable no 3: playing the keyboard with the MIROR-Impro with reflexive reply. The aim is to observe if the reflexive interaction using MIROR-Impro is *necessary* and/or *sufficient* to improve the dependent variables.

The dependent variables are as follows: (1) the children’s ability to improvise in solos, and (2) the children’s ability to improvise in duets.

### Procedure

The activities were carried out in a primary school. Several preliminary meetings were held with the teachers in order to introduce the MIROR Project, to discuss and share any issues, and to introduce and clarify the roles of the researchers and operators and the role of the teachers. A preliminary meeting was held with the children in order to assure a good relationship and positive attunement between researchers and children. The children were invited to participate freely. The observations were carried out in the school setting and the experimental activities were introduced in the daily routines of the children. The activities were carried out in a space in front of the class, so that the teacher could be present. Each child carried out a preliminary individual session to familiarize her/himself with the keyboard, the equipment, and the setting. This preliminary session lasted about 10 min for each child. After that, each child carried out the training activities (see the package of activities below), and then the Solo and the Duet tests. All sessions were video recorded.

Setting: for the training activities, three locations were prepared, one for each child. The children were accompanied two or three at a time to their personal location. Each location included a keyboard connected to a computer and provided with headphones. Each location was equipped with a video camera for video recording. The camera was attached to the keyboard in order to record the audio while the child listened through headphones. The child had the headphones in order to listen to her/his own productions, the productions of the system and, in the Duet task, those of the companion. In the Duet task, the setting included two keyboards and only one camera video-recorded both children. An assistant researcher managed the cameras and took note of the participants (names, ID number); the music teacher managed the keyboards and computers, and gave instructions to the children.

#### Package of Activities

Each child carried out a training program consisting of 5 weekly individual 12 min sessions. The CG played the complete package of independent variables (v1+v2+v3); EG1 played the keyboard and the keyboard with the MIROR-Impro with not-reflexive reply (v1+v2); EG2 played only the keyboard with the MIROR-Impro with the reflexive reply (v3).

The package of activities was organized as follows:

• *Control Group* (Complete package of activities): task 1- the child plays the keyboard (v1) (4 min); task 2 – the child plays the keyboard using the MIROR-Impro without reflexive reply (v2) (4 min); task 3 – the child plays the keyboard using MIROR-Impro with reflexive reply (v3) (4 min).
• *Experimental Group 1* (Package minus independent variable): task 1 – the child plays the keyboard (v1 = 6 min); task 2 – the child plays the keyboard using the MIROR-Impro without reflexive reply (v2) (6 min).
• *Experimental Group 2* (Independent variable minus Package): task 3 – the child plays the keyboard using MIROR-Impro with reflexive reply (v3) (12 min).

The different amount of time for each variable in each group is due to the fact that the convergence procedure is not used to verify which of the three independent variables considered is more effective, but rather to focus on only one variable which is manipulated in order to verify if the focused variable is sufficient and/or necessary for improving the dependent variable.

At each session, the child was asked to carry out the following tasks/games:

*Control Group*: “You can play three different games. One game is to play just the keyboard, another game is to play the keyboard with the system that will reply differently, another game is to play the keyboard with the system that will reply similarly. Each game lasts 4 min. After every 4 min we will ask you to change game. After 12 min we will ask you to stop. If you are tired you can stop and re-start when you want.”

*Experimental Group 1*: “You can play two different games. One game is to play just the keyboard; another game is to play the keyboard with the system that will reply differently. Each game lasts 6 min. After every 6 min we will ask you to change game. After 12 min we will ask you to stop. If you are tired you can stop and re-start when you want.”

*Experimental Group 2*: “You can play one game. You can play the keyboard with the system that will reply similarly. The game lasts 12 min. After 12 min we will ask you to stop. If you are tired you can stop and re-start when you want.”

In the first session the task order was chosen by the child. In the following sessions, the music teacher set the task order in a balanced manner. All sessions were video recorded.

#### Test

After doing the package activities, all participants of the three groups were submitted to a test. The test consisted of two tasks: the Solo task (the child improvises alone) and the Duet task (the children improvise in pairs).

**FIGURE 3:**
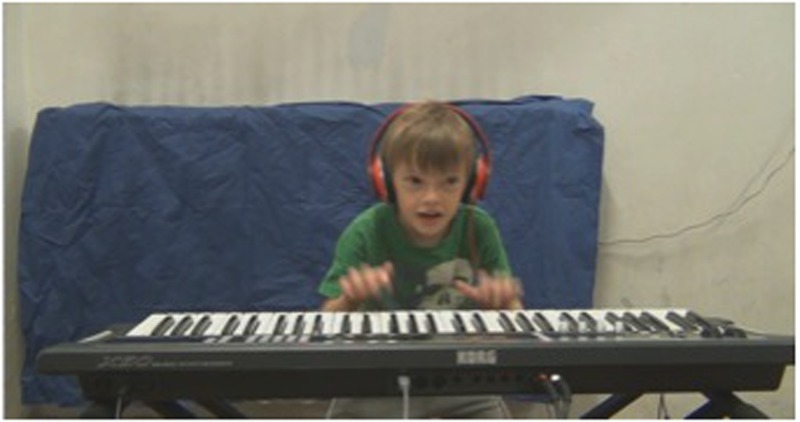
**An example of the Solo task setting**.

Each participant received the same instructions as follows:

*Solo Task*: “Create a short piece of music with a beginning, a development, and an end (like a story). You have 5 min to explore and invent the piece. After 5 min we will stop you and you will play your musical piece.” Before starting the performance, the music teacher repeats: “Now you play the musical piece that you invented. It should have a beginning, a development, and an end. When you finish, you stop and I understand that the piece is finished.” See **Figure 3** for an example of the Solo task setting.

During the 5 min available, the children had the possibility to explore some musical ideas, but did not write what they had created. Then, during the test, they performed their improvisation, in the sense that they elaborated in real-time the musical ideas previously explored (Kratus, 1996; McPherson, 2005; Tafuri, 2006). In the words of McPherson (2005, p. 10) they created “music aurally without the aid of notation”.

**FIGURE 4:**
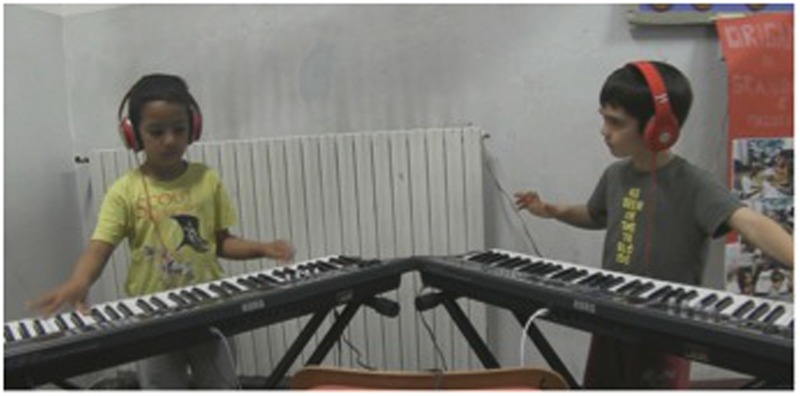
**An example of the Duet task setting**.

*Duet task*: “You can play the keyboards like in a dialog. In this dialog you can only use sounds, and not words. You have 10 min. At the end of the time, we will stop you.” See **Figure 4** for an example of the Duet task setting.

All sessions were video recorded.

In accordance with Campbell and Heller (1979), and Fiske (1992), we then compared:

(1) The results of the CG (“complete package” condition, v1+v2+v3) with the results of the Experimental Group 1 (“package minus” condition, v1+v2) to verify if the independent variable 3 (reflexive interaction using MIROR-Impro) is *necessary* to improve the dependent variables, that is the ability to improvise alone and in a duet: if CG > EG1 = independent variable 3 is *necessary* to improve dependent variables;
(2) The results of the CG (“complete package” condition”(v1+v2+v3) with the results of the Experimental Group 2 (“v2 minus package” condition, v3) to verify if the independent variable 3 (reflexive interaction using MIROR-Impro) is *sufficient* to improve the dependent variables, that is the ability to improvise alone and in a duet: if CG < EG2 = independent variable 3 is *sufficient* to improve dependent variables.

**Table 1** shows a synthesis of the experimental design.

**Table 1:** The convergence procedure (Campbell and Heller, 1979; Fiske, 1992), based on three sample groups (Control Group, Experimental Groups 1 and 2), allowing to verify if the reflexive interaction using the MIROR-Impro is *necessary* and/or *sufficient* to improve the children’s abilities to improvise, in solo and in duet.

Timeline	Control Group activity (complete package)	Experimental Group 1 activity (package minus v3)	Experimental Group 2 activity (v3 minus package)
5 weekly 12-min individual sessions	v1: playing only keyboard (4 min.) v2: keyboard with not reflexive reply (4 min.) v3: keyboard with MIROR reflexive reply (4 min.)	v1: playing only keyboard (6 min.) v2: keyboard with not reflexive reply (6 min.)	v3: playing keyboard with MIROR reflexive reply (12 min.)
1 week after: Test	Solo task Duet task	Solo task Duet task	Solo task Duet task

*Test results: if CG > EG1 = independent variable 3 (reflexive interaction using the MIROR-Impro) is necessary to improve dependent variables; if CG < EG2 = independent variable 3 is sufficient to improve dependent variables.*

#### Interview with the Children and Questionnaire to the Teacher

Each child was interviewed in order to collect data about their musical activities (e.g., playing an instrument). Moreover, a questionnaire was given to the teacher in order to collect information about the classroom activities done during music education lessons.

### Data Collected

The following data were collected:

• 47 individual preliminary sessions
• 47 × 5 individual sessions of the package activities
• Solo task: 47 individual improvisations lasting about 2 min each.
• Duet task: 23 duet improvisations lasting about 10 min each. Three cases were missed because of technical problems with the audio recordings and the setting. The following total number of duets were analyzed: eight duets with CG, six duets with Experimental Group number 1, six duets with Experimental Group number 2.

### Data Analysis

#### Assessment of Solo and Duet Tasks

Three independent judges assessed the Solo and Duet task improvisations by means of a grid based on the TAI (Test for Ability to Improvise, by McPherson, 1993, 1995, 2005) rating scale. The TAI is one of the three measures implemented by McPherson to assess high school instrumentalists’ ability to perform music (the other areas are the TAPFM-Test for Ability to Play From Memory, and the TAPE-Test for Ability to Play by Ear). In order to assess the ability to improvise (TAI), the students are asked to continue and conclude a musical sentence (“stylistically conceived” task) and to improvise a piece on their own that has a beginning, a middle, and an end (“freely conceived” task). The TAI includes four evaluative criteria: Instrumental Fluency (including Technical skill, Musical expression, and Musical fluency), Creativity (including Musical flexibility and Musical originality), Musical Syntax (including Ability to define style and Conception of logical response), and Musical Quality (see also McPherson, 1993).

Because the TAI was designed for high school instrumentalists, whereas in our study the participants were musically untrained children aged 6–7, it was necessary to introduce several modifications in order to adapt the TAI to the age and training level of the participants. Firstly, we only used the “freely conceived” task because the children were musically untrained and were at the beginner stage of musical learning, so they would not have been able to improvise on the basis of a particular musical style, as requested by the “stylistically conceived” task; for the same reason, the “Ability to define style” was excluded from the evaluative criteria of Musical Syntax: only the “Conception and logical response” was used, and we called this criterion Musical Organization. In conclusion, the evaluation grid of the study introduced in this article used the following four evaluative criteria: Instrumental Fluency, Creativity, Musical Organization, and Musical Quality. Some musical details were added for each criterion.

One other difference concerns the Duet task, because the TAI only foresees a solo improvisation task. Three new evaluative criteria were therefore added for the assessment of the Duet task, dealing with the ability of children to improvise in pairs: Musical Dialog, dealing with the children’s ability to dialog with a friend by means of sounds; Reflexive Interaction, dealing with the children’s ability to use reflexive interaction (repetition/variation, turn-taking, and co-regulation); and Attention Span, which is the duration of the dialog between the two children. In fact, some children stopped playing together and continued to play alone. Therefore, the judges were asked to indicate the time when the dialog between the children ended.

Finally, because the judges were asked to evaluate video recordings, instead of audio recordings as in the original TAI, they were also asked to consider the children’s bodily movement and expression. In accordance with several experts in children’s musical improvisation (for example: Delalande, 1993; Kratus, 1996; Mialaret, 1997; Sundin, 1998; Imberty, 2005), we considered the children’s musical improvisation as an experience of production/listening in which the gesture is fundamental, for both the sound quality of the product, and the child’s musical experience. In our specific case, for example, if the improvisation presented a repeated note, it was important for the judges to observe if the child was playing always with the same finger, or with several fingers, or with a lighter or harder touch, and observe the child’s facial expressions, her/his tension of the arms, the body position, and so on. The observation of the motor behaviors was also crucial in the evaluation of the Duet test, particularly, but not exclusively, for the Quality of Musical Dialog: the children’s motor behavior, for example, helped the judges to evaluate whether the child was stopping because she/he wanted to give the turn to her/his partner, or if she/he was tired of playing. In our study, the musical improvisation was considered an act of “production,” including motor and bodily gestures, which is not the same as in the tradition of music theory studies, where the object of analysis is primarily the sound or the scores.

The judges of our study were three professors of music education at an Italian university and/or conservatory, all experts in children’s music education. They worked together over several sessions. They first evaluated the Solo task and then the Duet Task. The Solo and the Duet tasks assigned to the children were communicated to the judges. The judges were required to watch the videos and to evaluate the children’s musical performances using a 5-point scale (the same as used by McPherson, 1993, 2005). For the Attention Span, the judges were asked to indicate when the dialog between the children ended (time in seconds). The judges were given an observation grid with the definitions of each category to be evaluated. They received the following written instructions: “Circle a number from 1 to 5 that indicates your rating for each of the assessed categories. Use the *Flow Chart of Evaluative Dimensions* (as in McPherson, 1993) in order to define the assessment criteria. You may use the observation of the child’s body movements, expressions, and gestures in order to better assess each category. Judgments should be made relative to one another and not according to absolute criteria. You may go back to the items already assessed and change your assessment. The videos can be watched as often as necessary, until each judge is satisfied with her/his assessment. Where appropriate, the videos can be stopped and re-watched if any of the judges want to re-hear/watch any performance. Generally the first 5–7 examples of the total performances should be used as consensus items, and to become familiar with the scoring methods for that particular item. The normal procedure then involves alternating between scoring up to five items in a row independently, followed by using another three or four items as consensus items. Where there is a break in the scoring, the judges again use the consensus approach adopted for the first three items at the commencement of the next session.” The consensus items were used to become familiar with the evaluative criteria and to check that, during the procedure (which lasted several sessions of 5/4 h each), the evaluative criteria were always clear and applied by all the judges in a coherent way. The consensus items were therefore used by the judges in order to discuss and clarify any conflicts of interpretation and application of the criteria. Having reached a consensus on the evaluative criteria, each judge gave her/his individual score to the item. For the Duet task, the following sentence was added at the beginning of the Instructions: “In this questionnaire you will be asked to assess the ability to improvise of two children who are each playing a keyboard.”

The judges received the following evaluative criteria used for both Solo and Duet tasks (see McPherson, 1993, pp. 139–141):

*Instrumental Fluency*: Ability to execute musical ideas clearly and accurately. This includes the ability of the child (children) to respond freely to musical ideas and to perform with technical skill and musical expression. The ability is demonstrated in the extent to which the child (children) can perform in a spontaneous manner, moving easily from one musical idea to another.^1^

*Musical Organization*: Ability to organize musical material in a freely conceived idiom. The task of the child (children) is to provide a performance that is inherently logical and which makes musical sense. Musical organization is demonstrated in the degree to which the improvisation demonstrates one or more organizational principles such as: imitation, repetition, variation, contrast, alternation, question/answer, beginning/development/closing, phrasing, etc.

*Creativity*: Ability to think divergently, as demonstrated in an original and imaginative product. This is evaluated through an analysis of: (1) *Musical flexibility*: the extent to which the child (children) can generate differing musical ideas, and manipulate/elaborate these ideas during the course of the improvisation. (2) *Musical originality*: the extent to which the child (children) can provide a musically unique or unusual response. A unique or unusual response may result from the manipulation and/or elaboration of pitch (e.g., use of sequence, etc.), or rhythm (e.g., diminution, augmentation, dotted vs. no dotted, metric vs. syncopated, etc.), or other musical elements (e.g., timbre, articulation, dynamics, etc.).

*Musical Quality*: (Overall Musical Appeal): Ability to fluently perform creatively conceived material in a freely conceived idiom. This is a global rating indicating your assessment of the overall musical appeal of the improvisation. It should indicate the extent to which a committed performance played expressively and in a musically meaningful and creative manner, was achieved.

*Free comments*: After completing the assessment, please write your free comment about the performance: write everything you found interesting and meaningful.

Furthermore, for the Duet task the judges also received the following three categories (which are not included in McPherson’s TAI test):

*Quality of Musical Dialog*: Ability to dialog and interact with the partner by using the sounds: paying attention to the musical proposal (listening), the ability to reply in a way correlated to the friend’s musical proposal (e.g., by repetition, variation, contrast, etc.), the presence of symmetries, co-regulation, sharing and co-production of musical ideas, the ability to show a global intentionality to dialog with the friend.

*Reflexive Interaction*: Ability to interact using repetition and variation, turn taking, and co-regulation.

*Attention Span*: The subjects’ tendency to persist in their contact with the activities, in this case the musical dialog with the other child, irrespective of any underlying aim. In fact, in some performances you can observe that after a while, the children start to play by themselves. This implies that their attention toward the musical dialog with the friend has ended. In this case, indicate the duration (in seconds) of the dialog, from the beginning of the performance to the moment when the children start to play by themselves.

In accordance with McPherson (2005), the evaluative criteria of Instrumental Fluency, Musical Organization, Creativity, and Musical Quality were considered equally important. In the case of the Duet Task, we also considered Quality of Musical Dialog equally important, on the basis of various perspectives on musical improvisation, which focused on the ability of the performers to dialog together (cfr. Sawyer, 2001; Gratier, 2008). The Reflexive Interaction was measured in order to verify if the use of the reflexive system “teaches” the children to use reflexive interaction during their improvised musical dialogs, and if there is some correlation with the ability to improvise.

**Table 2** shows the evaluation grid used by the judges.

**Table 2:** Evaluation grid used by the judges to assess the children’s musical performance.

	Hesitant and Labored				Spontaneous and Confident
Instrumental Fluency	1	2	3	4	5

	Illogical				Logical
**Musical Organization**	1	2	3	4	5

	No Uniqueness				Marked Uniqueness
**Creativity**	1	2	3	4	5

	Unappealing				Appealing
**Musical Quality**	1	2	3	4	5

	Little Dialog				Much Dialog
**Musical Dialog**	1	2	3	4	5

	Poor Reflexivity				Much Reflexivity
**Reflexive Interaction**	1	2	3	4	5

*To evaluate the SOLO task the judges only used Instrumental Fluency, Musical Organization, Creativity and Musical Quality. To evaluate the Duet task the judges used all criteria.*

#### Data Analysis

After the scoring, one score for each of the behaviors was calculated for each child for each task (Solo and Duet). First, each final score of each behavior was obtained by averaging the three scores registered by the three judges for each behavior. Then the level of ability to improvise was obtained by averaging the means of the behaviors, that is, for the Solo task, Instrumental Fluency, Musical Organization, Creativity, and Musical Quality, and for the Duet task, also the Quality of Musical Dialog. The Attention Span was calculated by averaging the duration of the dialog between the children of each duet and the correlation with each behavior was calculated. We calculated the correlation between Reflexive Interaction and each group, and between Reflexive Interaction and the overall ability to improvise. The final scores were considered for the statistical analyses. In order to evaluate the effect of the different conditions we performed planned pair-wise comparisons between the CG and Experimental Group 1 (EG1); and between the CG and Experimental Group 2 (EG2). Furthermore, we performed the same comparisons between EG1 and EG2. This was done separately for all behaviors including the overall ability to improvise, the Reflexive Interaction, and the Attention Span in the Duet task. We used independent-samples *t*-test assuming equal variances, which were computed using the SPSS software. Considering the relatively large number of independent comparisons (10 in the Solo task, 16 in the Duet task) it may have been appropriate to compensate for family wise error. However, in this case, the different behaviors were not considered independent. This was also confirmed in the measurements. Therefore, a compensation for family wise error, for example using Bonferroni correction, was considered to be a too conservative estimation. The reliability of the mean estimation across judges was assessed using Cronbach’s alpha. The judges’ agreement was estimated by the average Pearson’s correlation between all pairs of judges.

## Results

Below we report the results of the Solo and Duet tasks.

### Solo Task

#### Agreement of the Judges

Both Cronbach’s alpha and Pearson’s correlation between all pairs of judges indicate high agreement, as shown in **Table 3**. The Cronbach’s alpha was around 0.95, which is considered excellent according to a common rule-of-thumb. Thus, it indicates that the calibration procedure among the judges was efficient.

**Table 3:** Solo task: Agreement of the judges.

	Cronbach’s alpha	pairwise corr
Instrumental Fluency	0.93	0.83
Musical Organization	0.96	0.88
Creativity	0.95	0.86
Musical Quality	0.95	0.86
**Mean**	**0.95**	**0.86**

#### Total Score of the Solo Task

**FIGURE 5:**
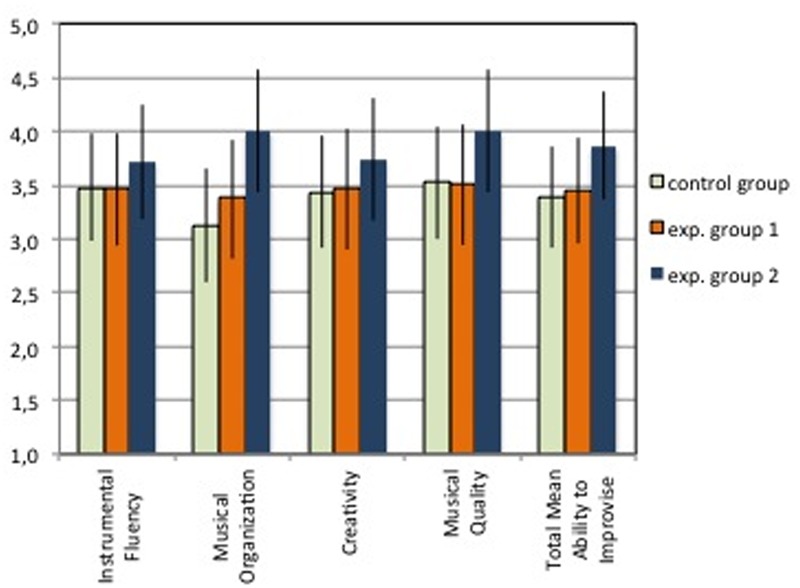
**Solo task: Scores for each evaluative criteria and the Total score of Ability to Improvise (means).** The error bars indicate 95% confidence intervals.

The total score of the Solo task (see **Table 4**; **Figure 5**) shows that the CG (3.39), which trained with the complete package of variables, that is to play only with the keyboard, with the reflexive system, and with the not reflexive system, obtained a slightly lower score compared to the EG1 (3.46), which did not play with the reflexive system. Following the experimental design, this result indicates that the independent variable of reflexive interaction (v3) may *not be necessary* to improve the dependent variable of ability to improvise (CG > EG1). The results also show that the CG obtained a lower score compared to the EG2 (3.86), which only played with the reflexive system. In this case, it could be said that the reflexive interaction could be *sufficient* to improve the ability to improvise (CG < EG2). However, it is not possible to generalize these results because the differences were not statistically significant (*t*-test, comparing CG and EG1, *p* = 0.854; CG and EG2, *p* = 0.198).

**Table 4:** Solo task: Score for each evaluative criteria (Instrumental Fluency, Musical Organization, Creativity, and Musical Quality), and the Total score of Ability to Improvise (means).

	Instrumental Fluency	Musical Organization	Creativity	Musical Quality	Total score of Ability to Improvise
Control Group	3.48 (±0.50)	3.13 (±0.53)	3.44 (±0.53)	3.52 (±0.53)	3.39 (±0.47)
Experimental Group 1	3.47 (±0.51)	3.38 (±0.54)	3.47 (±0.55)	3.51 (±0.55)	3.46 (±0.49)
Experimental Group 2	3.71 (±0.53)	4.00 (±0.56)	3.74 (±0.57)	4.00 (±0.57)	3.86 (±0.51)
*CG^∗^EG1* *CG^∗^EG2*	*p* = 0.970 *p* = 0.547	*p* = 0.527 *p* = 0.039	*p* = 0.942 *p* = 0.452	*p* = 0.981 *p* = 0.221	*p* = 0.854 *p* = 0.198
*EG1^∗^EG2*	*p* = 0.499	*p* = 0.080	*p* = 0.453	*p* = 0.184	*p* = 0.207
	*CG* > *EG1* *necessary*	*CG* < *EG1* *not necessary*	*CG* < *EG1* *not necessary*	*CG* > *EG1* *necessary*	*CG* < *EG1* *not necessary*
	*CG* < *EG2* *sufficient*	*CG* < *EG2* *sufficient*	*CG* < *EG2* *sufficient*	*CG* < *EG2* *sufficient*	*CG* < *EG2* *sufficient*

*Numbers in parenthesis are 95% confidence intervals (means). According to the experimental design if the score of the Control Group is higher than the Experimental Group 1, then the independent variable of reflexive interaction with MIROR-Impro (v3) is *necessary* to improve the ability to improvise in solo performance; if the score of the Control Group is lower than the Experimental Group 2, then the independent variable reflexive interaction with MIROR-Impro (v3) is *sufficient* to improve ability to improvise in solo performance.*

#### Results for Each Evaluative Criterion of the Solo Task

The analyses on each evaluative criterion confirm the overall trend (**Table 4**; **Figure 5**). Regarding the condition of “sufficiency,” the comparison between EG2, which only played with the reflexive system, and the CG, which also played with the not-reflexive system and only with the keyboard, shows that EG2 reaches higher scores in all evaluative criteria. This result shows, therefore, that the use of the reflexive system alone could be *sufficient* to improve both Instrumental Fluency, Musical Organization, Creativity, and Musical Quality. Regarding the condition of “necessity,” the comparison between EG1, which never played with the reflexive system, and the CG, which also played with the reflexive system, shows that the score of EG1 is slightly lower in Instrumental Fluency and Musical Quality and slightly higher in Musical Organization and Creativity. These results show that, for the sample of participants, the use of the reflexive system could be *necessary* to improve Instrumental Fluency and Musical Quality in solo improvisation, but *not necessary* to improve Musical Organization and Creativity. However, as indicated by the *p*-values in **Table 4**, the differences between the CG and the Experimental Groups 1 and 2 are rarely significant and thus it is problematic to generalize these results. The only exception was Musical Organization that was significant and received a *p*-value of 0.039 comparing CG with EG2.

Moreover, it is worth noting that EG2, the group that trained only with the reflexive system, showed a higher score in all criteria.

**FIGURE 6:**
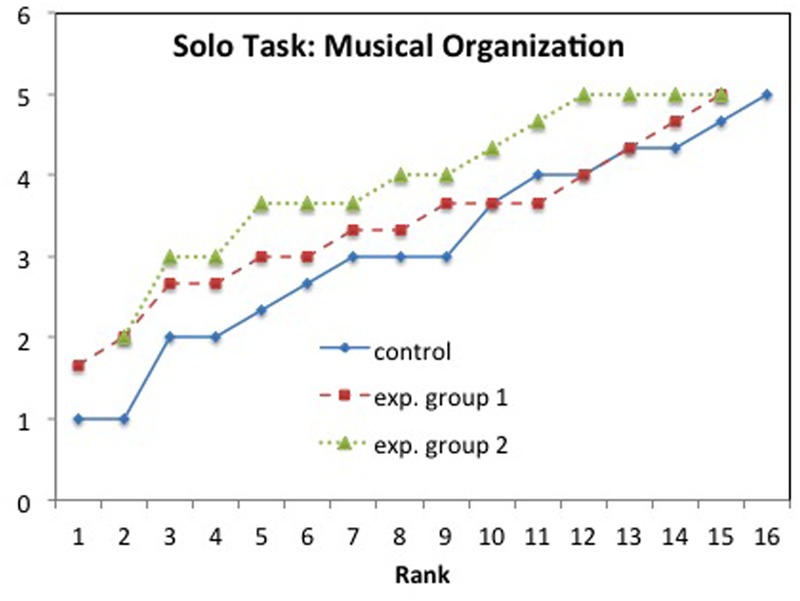
**Solo task, *Musical Organization*: Mean values rank-ordered in each group**.

In order to examine the differences between the groups in more detail we plotted each child rank-ordered within each group, see **Figure 6**. It is clear from the figure that the variation within each group is comparatively large but also that there is a (smaller) difference between the groups, in particular between CG and EG2.

### Duet Task

#### Agreement of the Judges

Both Cronbach’s alpha and Pearson’s correlation between all pairs of judges indicate high agreement, as shown in **Table 5**. The Cronbach’s alphas were in this case also excellent and even higher than for the solo task, and for two behaviors reached 0.99.

**Table 5:** Duet task: agreement of the judges.

	Cronbach’s alpha	pairwise corr
Instrumental Fluency	0.94	0,84
Musical Organization	0.97	0.91
Creativity	0.98	0.94
Musical Quality	0.96	0.91
Musical Dialog	0.99	0.97
Reflexive Interaction	0.99	0.97
**Mean**	**0.97**	**0.92**

#### Total Score of the Duet Task

**FIGURE 7:**
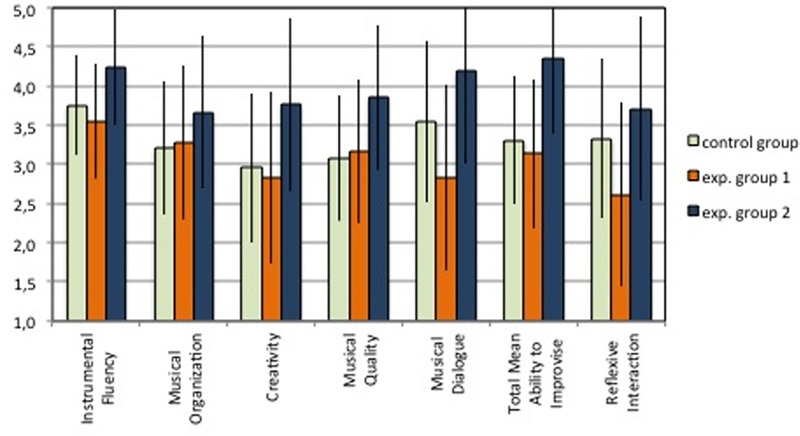
**Duet task: Scores for each evaluative criteria, Total score of Ability to Improvise, and score of Reflexive Interaction (means).** The error bars indicate the 95% confidence intervals.

The total score of the Duet task shows that the CG (3.31), which played the complete package of variables including the reflexive system, obtained a higher score compared to the EG1 (3.05), which did not play with the reflexive system (**Table 6**; **Figure 7**). Therefore, the total trend indicates that for the sample of participant the reflexive interaction using the MIROR-Impro (variable 3) could be *necessary* to improve children’s ability to improvise. Comparing the CG with the EG2 (4.32), which only played with the reflexive system, the CG obtained a lower score. Therefore, the total trend indicates that for the sample of participants the reflexive interaction using the MIROR-Impro (variable 3) could be *sufficient* to improve children’s ability to improvise. However, it is not possible to generalize these results because the differences are not statistically significant (t-test, comparing CG and EG1, *p* = 0.707; CG and EG2, *p* = 0.124).

**Table 6:** Duet task: Score for each evaluative criteria (Instrumental Fluency, Musical Organization, Creativity, Musical Quality, and Musical Dialog), the Total score of Ability to Improvise, and the score of Reflexive Interaction (means).

Group	Instrumental Fluency	Musical Organization	Creativity	Musical Quality	Musical Dialog	Total score of Ability to Improvise	Reflexive Interaction
CG	3.75 (±0.64)	3.21 (±0.85)	2.96 (±0.96)	3.08 (±0.80)	3.54 (±1.03)	3.31 (±0.82)	3.33 (±1.02)
EG1	3.56 (±0.74)	3.28 (±0.98)	2.83 (±1.10)	3.17 (±0.93)	2.83 (±1.19)	3.13 (±0.95)	2.61 (±1.17)
EG2	4.50 (±0.74)	4.11 (±0.98)	4.22 (±1.11)	4.33 (±0.93)	4.56 (±1.19)	4.34 (±0.95)	4.17 (±1.18)
*CG^∗^EG1* *CG^∗^EG2*	*p* = 0.702 *p* = 0.135	*p* = 0.922 *p* = 0.164	*p* = 0.877 *p* = 0.087	*p* = 0.898 *p* = 0.063	*p* = 0.427 *p* = 0.126	*p* = 0.799 *p* = 0.101	*p* = 0.388 *p* = 0.285
*EG1^∗^EG2*	*p* = 0.059	*p* = 0.152	*p* = 0.046	*p* = 0.039	*p* = 0.043	*p* = 0.047	*p* = 0.043
	*CG* > *EG1* *necessary*	*EG1* > *CG* *not necessary*	*CG* > *EG1* *necessary*	*CG* > *EG1* *necessary*	*CG* > *EG1* *necessary*	*CG* > *EG1* *necessary*	*CG* > *EG1* *necessary*
	*CG* < *EG2* *sufficient*	*CG* < *EG2* *sufficient*	*CG* < *EG2* *sufficient*	*CG* < *EG2* *sufficient*	*CG* < *EG2* *sufficient*	*CG* < *EG2* *sufficient*	*CG* < *EG2* *sufficient*

*Numbers in parenthesis are 95% confidence intervals. According to the experimental design if the score of the Control Group is higher than the Experimental Group 1, then the independent variable of reflexive interaction with MIROR-Impro (v3) is *necessary* to improve the ability to improvise in duet; if the score of the Control Group is lower than the Experimental Group 2, then the independent variable reflexive interaction with MIROR-Impro (v3) is *sufficient* to improve ability to improvise in duet.*

#### Results for Each Evaluative Criterion of the Duet Task

The analyses on each criterion of the Duet task confirm the total trend, with few exceptions; see **Table 6** and **Figure 7**. Regarding the condition of “sufficiency,” the comparison between EG2, which only played with the reflexive system, and the CG, which also played with the not reflexive system and only the keyboard, shows that the EG2 reaches higher scores in all evaluative criteria. This trend shows, therefore, that the use of the reflexive system alone could be *sufficient* to improve all behaviors. Regarding the condition of “necessity,” the comparison between EG1, which never played with the reflexive system, and the CG, which also played with the reflexive system, shows that the score of EG1 is lower in all criteria, excluding Musical Organization and Musical Quality. This trend shows that the use of the reflexive system could be *necessary* to improve Instrumental Fluency, Creativity, and Musical Dialog. Also in this case it is not possible to generalize these results because the *p-*values are not significant. However, note that the trend is close to significance with Musical Quality, in the *necessity* condition, (CG = 3.08; EG2 = 4.33; *p* = 0.063). It is important to note that, also in the Duet Task, EG2, which played just with the reflexive system, shows a higher score in all criteria in comparison with both CG and EG1. The difference between EG1 and EG2 is statistically significant (*p* < 0.05) for Creativity, Musical Quality, Musical Dialog, and for the overall ability to improvise, and close to the significance for Instrumental Fluency (*p* = 0.059).

**FIGURE 8:**
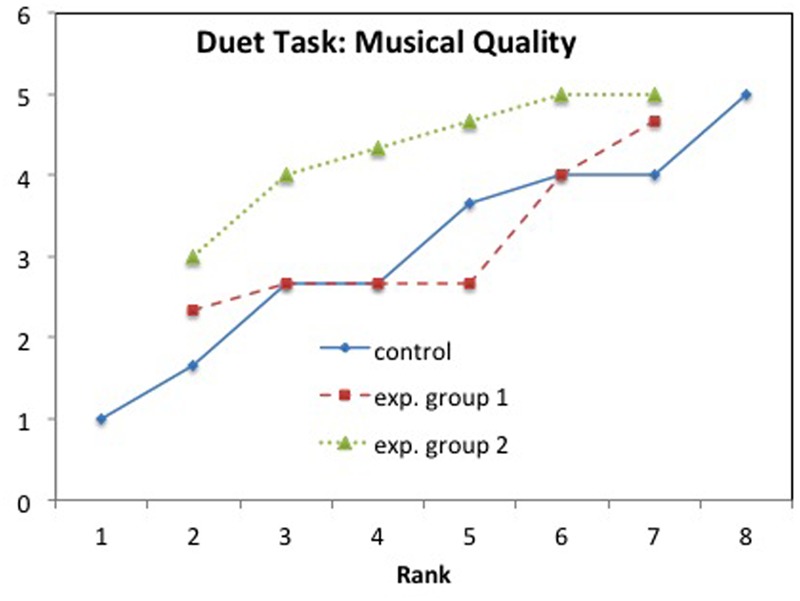
**Duet task: Mean values rank-ordered in each group for Musical Quality**.

**Figure 8** gives the mean values rank-ordered for each group with Musical Quality, showing that the EG2 score is always above that of the other two groups. Note that the variation within groups is rather large and represents a possible source for the non-significant results between EG2 and CG.

##### Reflexive Interaction

The data of Reflexive Interaction show that the EG2 obtained the highest score (4.17), followed by the CG (3.33) and the EG1 (2.61); see **Table 6** and **Figure 7**. The difference between EG1, which only use the system with reflexive interaction, and EG2, which did not use the system with reflexive interaction, is significant (*p* = 0.043). Therefore, it could be said that the use of MIROR-Impro can enhance the use of the reflexive behaviors: mirroring, turn-taking, and co-regulation. We observed a statistically significant correlation between the Reflexive Interaction and the total score (*r* = 0.937; *p* < 0.01), and all other evaluative criteria, with correlations ranging from *r* = 0.87 (*p* < 0.01) for Musical Quality to *r* = 0.92 (*p* < 0.01) for Musical Organization. Thus, the higher the children’s use of reflexive interaction, the better their results in each criterion and in the ability to improvise. This result can support the hypothesis that reflexive interaction is a fundamental component of musical improvised dialog. Instead, although the differences between the CG and the Experimental Groups 1 and 2 indicate that the use of the MIROR-Impro appears to be “necessary” (CG > EG1) and “sufficient” (CG < EG2) to improve the ability to improvise, we cannot generalize these results because the results are not statistically significant (*t*-test, comparing CG and EG1: *p* = 0.388; CG and EG2: *p* = 0.285).

##### Attention Span

**FIGURE 9:**
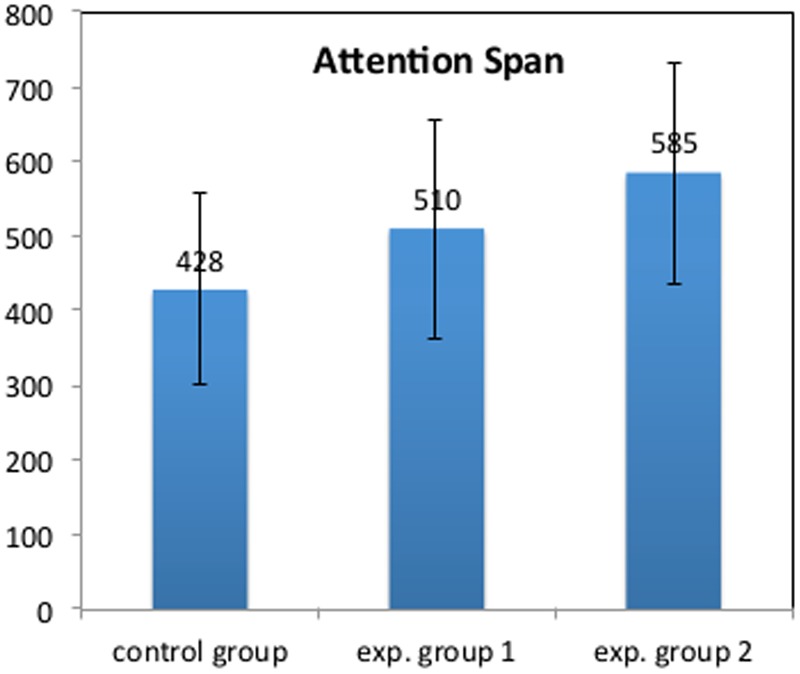
**Duet task: Plot of the attention span in each group.** The columns indicate the duration of children’s attention in seconds. The error bars indicate the 95% confidence intervals.

The Attention Span indicates the subjects’ tendency to persist in their contact with the activities, in this case the musical dialog with the other child, irrespective of any underlying aim. In fact, in some performances, after a while, the children started to play by themselves. This means that their attention for the musical dialog with the friend had ended. The judges were asked to indicate the duration (in seconds) of the dialog, from the beginning of the performance to the moment when the children start to play by themselves. The results show that the EG2 (585 s), which trained only with the reflexive system, reaches the highest Attention Span, compared with the CG (428 s) and the EG1 (510 s) (see **Figure 9**). In particular, we observed a statistically significant correlation between the Attention Span and all other evaluative criteria, with correlations ranging from *r* = 0.52 (*p* < 0.05) for Instrumental Fluency to *r* = 0.63 (*p* < 0.01) for Musical Quality. Thus, the longer their attention span, the better their results in all criteria.

## Discussion

In this article, we introduced a study about the effects of reflexive interaction with the software MIROR-Impro on the musical improvisation ability of children playing a keyboard. In the framework of the MIROR project and, more in general, in the field of studies on reflexive technology and children, this is the first study which assesses in a controlled way the effects of reflexive technologies on the children’s ability to improvise. The study was conducted with 47 children aged 7 and 8 years, and used the experimental design of convergence procedure and the TAI-Test of Ability to Improvise (McPherson, 2005), partially adapted to the purposes of this study. Two conditions were observed: the condition of “sufficiency” (RI with MIROR-Impro is sufficient to develop the ability to improvise) and the condition of “necessity” (RI with the MIROR-impro is necessary to develop improvisational skills). The trend of the results with the sample of participants indicates that RI with MIROR-Impro alone can be *sufficient*, compared to the remaining two observed variables (playing only with the keyboard and playing with the same software but programmed with a non-reflexive response), to increase the improvisational skills of the subjects who participated in the study, both when improvising alone and in a duet. The trend also shows that RI using MIROR-Impro can be *necessary*, compared to the remaining two variables considered, to develop the ability to improvise in duets. However, the results of necessity and sufficiency are in general not statistically significant and they cannot be generalized. A greater number of subjects (to control the internal variance of subjects) and a longer training period (6 months rather than 5 weeks, and 1 h per session rather than 12 min), could have strengthened the experimental design, and then given a greater statistical significance. It is interesting to note that the trend is more evident in the Duet task, where some results and the correlation between Reflexive interaction and the ability to improvise are statistically significant or closer to the significance. The EG2, which trained only with the reflexive system, reached always the highest average results, both when the children improvise alone and in a duet. The difference between EG2 and EG1, which did not use the reflexive system, is statistically significant in the Duet task. Even though it was not part of the experimental design to test this, it indicates that the training with reflexive system was effective for the ability to improvise in pair. In conclusion, the results of the study are interesting and worth exploring the reflexive interaction paradigm further in the field of children’s musical improvisation, creativity and education.

### Reflexive Interaction, Musical Improvisation, and Creativity

In the Duet task, the behaviors characteristic of the Reflexive Interaction (mirroring, turn-taking, and co-regulation) were statistically correlated with the ability to improvise; furthermore, the children that used the interactive system alone during the training program showed the highest results for these behaviors. It seems that reflexive interaction using the MIROR-Impro enhances some processes of music dialog that the child learns during the interaction with the system and then uses even when the interaction occurs with a partner. Repetition in music takes on particular importance, both in improvisation and composition, as well as in listening, and concerns all aspects, from music analysis to the cognitive and neurobiological processes (cfr. Margulis, 2014). In the specific field of children’s musical experience, it was observed how the repetition-variation action during the explorations of sound objects in early childhood allow the child to know a sound, to share it with others, to invent music and express emotions (Delalande, 1993; Kratus, 1996; Baroni, 1997); in children’s musical improvisation with instruments, repetition and mirroring becomes the fundamental principle of invention (cfr. Young, 2004; Tafuri, 2006). In the practice of improvisation, the repetition and variation mechanism can structure the musical discourse over time, establishing the basic pillars for exploration and transformation, thereby paving the way for the creation of new musical ideas. Recent studies in neuroscience underline the neural and cognitive mechanisms that allow us to transform and manipulate existing musical representations. Zatorre (2012) suggests that the dorsal pathway of auditory processing performs equivalent operations on musical inputs. The results allow new hypotheses about how novel musical ideas may emerge from pre-existing musical images, by means of the mechanism of repetition and variation. This finding highlights the close link between improvisation, repetition and variation, and creative processes. Kleinmintz et al. (2014) points out that musical improvisation increases creativity. The link between improvisation and creativity was also emphasized in neurobiology, where it was found that the greater connectivity between brain regions sharing functional properties observed in professional improvisers may be due to a more efficient working of the associative networks of musical creativity (Pinho et al., 2014; see also Beaty, 2015). In our study, the Experimental Group 2, which only use the MIROR reflexive system, shows the highest score not only in Reflexive Interaction behaviors but also in the Creativity criterion. This result also reinforces our previous study, which showed the effectiveness of RI with MIROR-Impro in the development of creative processes, as described in the flow theory (Addessi et al., 2015).

Turn-taking is fundamental in reflexive interaction. Neurobiological studies have highlighted the close link between turn-taking and the processes of imitation and suggest that “simulation is a foundational mechanism underlying the temporal dynamics of joint action” (Hadley et al., 2015, p. 19516. Cfr. also Gratier et al., 2015). Another important mechanism of reflexive interaction is immediate feedback, which allows the child to have a perception of the relevance of its intervention in relation to that of the virtual partner. The importance of improvisation feedback is highlighted by Pressing when he writes “Feedback is a vital component in improvisation for it enables error correction and adaptation – a narrowing of the gap between intended and actual motor and musical effects“ Pressing (1988).

Therefore, the rules of reflexive interaction (turn-taking, listening to the partner, repetition and variation, co-regulation), become musical rules. These rules help the children to listen to and invent musical improvisation based on turn-taking (musical phrasing), repetition, variation, contrast, dialog, etc. Some of these mechanisms are indicated by several scholars to be the basis of creativity and improvisation: e.g., Sawyer (2001) introduces improvisation in everyday discourse; Berkowitz (2010) describes various kinds of variations, recombination and combinatory procedures as pedagogical strategies for teaching music improvisation. Since reflexive interaction develops rules of interaction, the results of our study also support the idea that the collective performance cannot only be studied from a purely musical point of view but also on the basis of the interactive and communicative context between the partners (Schiavio and Høffding, 2015), and on the basis of social interaction characteristics (e.g., Sawyer, 2001; Littleton and Mercer, 2012; Pesquita et al., 2014; Kawase, 2015). In this regard, Moran et al. (2015) highlighted that the back-channeling cues during a free improvisation do not dependent on specific music skills but rather on a general human ability to communicate. In particular, the results of the Duet task in our study could be an efficient index of the effects of reflexive interaction, thanks to the presence of several characteristics of the co-performer interaction (e.g., Williamon and Davidson, 2002; Seddon, 2005; Gratier, 2008; Moran et al., 2015).

### Child–Machine Interaction in a Reflexive Environment

In the field of child–machine interaction, the results of this study can acquire a particular interest for two reasons: the first is by presenting a new scientific paradigm, reflexive interaction, which proves to be empirically effective in developing creative and improvisational skills in children; the second concerns the type of methodology used, which started not from theoretical assumptions on child–machine interaction, but from the observation of the concrete and naturalistic context of such interaction. Our observation has given rise to hypotheses, empirical studies and theoretical explanations in the scientific literature. It has been possible to observe the child–machine interaction and note how RI develops between the child and the system without adult mediation. This issue is particularly important in the field of technology and children’s education, because the presence of the adult as mediator between child and machine is generally considered fundamental. This is not to say that reflexive systems should replace the teacher (although they could do so when the teacher does not want or is not able to teach improvisation), but rather that they can have an important role in learning to improvise and, in general, in enhancing children’s musical creativity. In the reflexive setting, the role of the teacher would be to create the context where the children, alone or in groups, can interact with the system, and to re-launch the more creative musical ideas of the children: in other words, to use the reflexive system as a “device,” in the meaning that Delalande (1993) gave to it, that is a tool to motivate and reinforce the musical creativity of children (see Addessi, 2015).

### Implication in Music Education

The results of this study contributes to the research on children’s musical improvisation, highlighting new details about children’s processes of musical improvisation. An important aspect of the results of this study is that they show how the reflexive interaction with the system also affects the intrinsic motivation of the child while playing. The results show in fact that the attention spans of children of Experimental Group 2, who have training only with MIROR-Impro, are higher than the Experimental Group 1 (which never played with MIROR-Impro) and the CG (which trained with MIROR-Impro with and without reflexive reply, and only the keyboard). This result, which confirms those of a previous study (Addessi and Pachet, 2006), shows that RI with MIROR-Impro develops intrinsic motivation, which is considered one of the most important conditions for learning (cfr. Austin et al., 2006). We consider it important to emphasize that the effectiveness of IR on the ability of improvisation derives from the fact that this develops an intrinsic motivation to participate in a musical dialog: children can express themselves by means of sounds, which is a fundamental need of children. As Baroni writes: “We believe it is possible to maintain a rigid position of principal, that is, the absolute necessity for the pre-eminence of expression over learning: and this is not only because the construction of expressive objects can be considered the principal goal, but also because it constitutes the only valid and persuasive motivation for learning activities” (1997, p. 141).

From a pedagogical point of view, we suggest that the child and the teacher can use the MIROR-Impro in order to support an improvisation teaching program by means of individual and collective “deliberate practice” (Ericsson, 1997; Kenny and Gellrich, 2002; McPherson, 2005) with the system. It is important in fact to emphasize that the training activities of our experiment were carried out as “deliberate practice”: the children trained by themselves, out of their own choice, during normal educational activities. Such systems may have a use in basic schooling as a means of expression, to dialog through sounds, to invent music and build relationships among peers (Cardoso de Araújo, 2015; Ferrari and Addessi, 2015), as well as helping music improvisation classes to learn/teach techniques of improvisation (exploration, imitation, invention, dialog with sounds, listening) (cfr. Benghi and Addessi, 2015; Nijs and Leman, 2015). As the interaction is based mainly on sound, this interaction could be very important for the development of the child’s *thinking in sound*, which has been identified as one of the most important aims for music education and music improvisation (cfr. Webster, 1994; Williamon, 2004; McPherson, 2005). They can also be used for teacher education to develop teachers’ basic musical competences (making music, improvisation and composition, listening), teachers’ professional competences related to child–machine interaction, children’s musical development, children’s musical creativity, and reflection about the role of teacher in reflexive environments. Finally, reflexive interaction can itself become a teaching technique, and the teachers can learn how to teach music improvisation: dialoguing with children by means of sounds, mirroring the musical ideas of children, using a repetition and variation approach, respecting turn-taking, co-regulating the dialog, etc.

### Future Directions

The results of this study, and the video analysis of the training activities, will contribute to create a list of technical and pedagogical requirements to improve the reflexive technology to support children’s ability to improvise: for example, the possibility to use special rhythmic-melodic patterns, or alternative setups (cfr. Regazzi and Benghi, 2015). The results can also contribute to better understand the reflexive interaction mechanism in human interactions, in particular in infant-adult interaction based on turn-taking and the mechanism of repetition-variation: with this aim, further studies are planned to observe the presence of reflexivity in infant-adult interaction. In the field of neurobiology, the reflexive technologies could represent an efficient tool to investigate the “resonance” (Rizzolatti et al., 2002) mechanism related to sound perception, and the neural and cognitive processes that allow us to transform and manipulate existing musical representations (Berkowitz, 2010; Zatorre, 2012). The midi data collected during sessions with the system could allow computational analysis of improvisation based on reflexive interaction. Some tentative steps have already been made (Anagnostopoulou et al., 2012; Alexakis et al., 2013) and this could also offer future perspectives on the basis of some positive experiences of computational analysis of musical dialog between therapist and patient (Luck et al., 2008; Erkkilä et al., 2012; Wigram, 2012). As we wrote, “the musical analysis of reflexive dialog should allow us to capture the aspects that characterize the presence of structural variation in repeated patterns, and to hypothesize how these variations are produced” (Addessi, 2014, p. 222). At this moment, the methodology of observation seems to be the most effective tool to study reflexive interaction in children’s musical improvisation, as it captures aspects of improvisational behavior linked to the gestures, body communication, and intentionality of the subjects, which cover the full experience of musical improvisation, as also underlined by a recent study in music performance (King and Gritten, 2016). In this regard, the TAI-Test of Ability to Improvise (McPherson, 1993, 2005), including the changes made for this study, has proved to be an effective and flexible research tool (cfr. Thompson and Williamon, 2003) for the assessment of children’s ability to improvise, not only for audio data, as in the original Test, but also for the video performance assessment. We are working in order to implement an observational grid for a more detailed analysis of the musical dialogs of the duets, also in collaboration with experts in computational video analysis and motion capture systems.

The reflexive interaction paradigm and reflexive technologies are relatively new in the field of music education and teaching improvisation. The MIROR project aimed to exploit this paradigm in the field of children’s music and movement creativity and education, in terms of technology (three applications were implemented: MIROR-Impro, MIROR-Compo and MIROR-Body Gesture), empirical and theoretical research, and pedagogical practices. The process of spiral implementation of this kind of technology has not yet finished: the MIROR applications can be perfected on the basis of the empirical experiences that will continue to be carried out by children, teachers, researchers, and therapists. In this context, the study introduced in this paper indicated that reflexive interaction and the reflexive technologies can have a positive effect on children’s ability to improvise and we suggest introducing this device in the program of teaching to improvise and as an exciting tool for children’s “deliberate practice.” A new longitudinal case-study has been planned to analyze the effects of reflexive interaction using the MIROR-Impro in deliberate practice, over a longer period of activities. The future challenge is to implement a new MIROR application based on reflexive interaction, called MIROR-MultiModal, which will also involve the visual perception of children, and to design and implement the MIROR platform, a learning/teaching environment with related architecture and technological tools, for children, teachers, and researchers.

## Ethics Statement

The study has been approved by the Legal Office of Research Department of the University of Bologna, which took care of the legal and ethical aspects related to the Consent form, and by the Primary School of Casalecchio di Reno (Italy). All the children’s parents gave their written informed consent in accordance with the relevant national, European and international data protection laws and regulations and personal data treatment obligations. Each judge signed a confidentiality undertaking. Specifically the consent form complied with the following laws and regulations:

• EC Data Protection Directive 95/46/EC of the European Parliament and of the Council of 24 October 1995 on the protection of individuals with regard to the processing of personal data and on the free movement of such data.
• Council of Europe Recommendation 83/10 Protection of personal data used for scientific research and statistics.
• Council of Europe Recommendation 97/18 Concerning the protection of personal data collected and processed for statistical purposes.
• Italian Legislative Decree No 196/2003.
• Attachment A4 to the Italian Legislative Decree No 196/2003 “Code of conduct and professional practice applying to processing of personal data for statistical and scientific purposes” (Published in the Official Journal no. 190 of August 14, 2004).

## Author Contributions

AA wrote the paper and is the principal investigator. AF collaborated in carrying out the experiment and in the statistical analysis. DB collaborated in the data collection. AF elaborated the statistical analyses and contributed in the corresponding text, figures, and tables. The final manuscript was supervised by all authors.

## Conflict of Interest Statement

The authors declare that the research was conducted in the absence of any commercial or financial relationships that could be construed as a potential conflict of interest.

## Footnotes

1 In accordance with the perspective of children’s spontaneous improvisation (Delalande, 1993; Kratus, 1996; Baroni, 1997; Webster, 2002; Tafuri, 2006), the term technical skills was not intended as indicating the ability to perform particular instructions, but rather the ability of the children to control the keyboard in a spontaneous way, for example to play in different registers, to use different gestures and combination of gestures (with one finger, with all fingers, one hand, two hands, symmetrically or with alternation of the gestures). According to this perspective in children’s improvisation a musical idea is, for example, a rhythmical pattern, or a sequence of sounds, preconceived or initially generated randomly, which is repeated and elaborated with variations.
