# Corrigendum: Child–Computer Interaction at the Beginner Stage of Music Learning: Effects of *Reflexive Interaction* on Children's Musical Improvisation

**DOI:** 10.3389/fpsyg.2017.00399

**Published:** 2017-03-31

**Authors:** Anna R. Addessi, Filomena Anelli, Diber Benghi, Anders Friberg

**Affiliations:** ^1^Department of Education Studies, University of BolognaBologna, Italy; ^2^Department of Speech, Music and Hearing, KTH Royal Institute of TechnologyStockholm, Sweden

**Keywords:** reflexive interaction, children's music improvisation, child-computer interaction, assessment of children's performance, MIROR-Impro

In the original article, there was an error. “she plays C3” was used instead of “it plays C3.”

A correction has been made to Observation and Theoretical Framework of Reflexive Interaction, paragraph 3:

*The little girl plays two consecutive notes, C2 and A2, and then stops to wait for the response of the system. The system responds by repeating the same notes. The child then play a single note, G2, and the system responds with a single note but this time introduces a variation: it plays C3, thus introducing a higher register. The girl, following the change introduced by the system, moves toward the higher register and plays a variant of the initial pattern, namely: D2-A2-E2-C3, and introduces a particular rhythm pattern. This “reflexive” event marks the beginning of a dialogue based on repetition and variation: the rhythmic-melodic pattern will be repeated and varied by both the system and the child in consecutive exchanges, until acquiring the form of a complete musical phrase. At some point in the dialogue, the child begins to accompany the system's response with arm movements synchronized with the rhythmic-melodic patterns, creating a kind of music-motor composition*.

In addition, EG1 and EG2 are incorrectly referred to within the text.

A correction has been made to Duet Task, sub-section Results for Each Evaluative Criterion of the Duet Task, paragraph *Reflexive Interaction*:

The data of Reflexive Interaction show that the EG2 obtained the highest score (4.17), followed by the CG (3.33) and the EG1 (2.61); see Table 6 and **Figure 7**. The difference between EG2, which only use the system with reflexive interaction, and EG1, which did not use the system with reflexive interaction, is significant (*p* = 0.043). Therefore, it could be said that the use of MIROR-Impro can enhance the use of the reflexive behaviors: mirroring, turn-taking, and co-regulation. We observed a statistically significant correlation between the Reflexive Interaction and the total score (*r* = 0.937; *p* < 0.01), and all other evaluative criteria, with correlations ranging from *r* = 0.87 (*p* < 0.01) for Musical Quality to *r* = 0.92 (*p* < 0.01) for Musical Organization. Thus, the higher the children's use of reflexive interaction, the better their results in each criterion and in the ability to improvise. This result can support the hypothesis that reflexive interaction is a fundamental component of musical improvised dialog. Instead, although the differences between the CG and the Experimental Groups 1 and 2 indicate that the use of the MIROR Impro appears to be “necessary” (CG > EG1) and “sufficient” (CG < EG2) to improve the ability to improvise, we cannot generalize these results because the results are not statistically significant (*t*-test, comparing CG and EG1: *p* = 0.388; CG and EG2: *p* = 0.285).

Finally, due to the resolution of Figures 5–9 being low, they have been replaced with new figures with a higher resolution. The corrected Figures, Figures 5–9 appear below.

**Figure 5:**
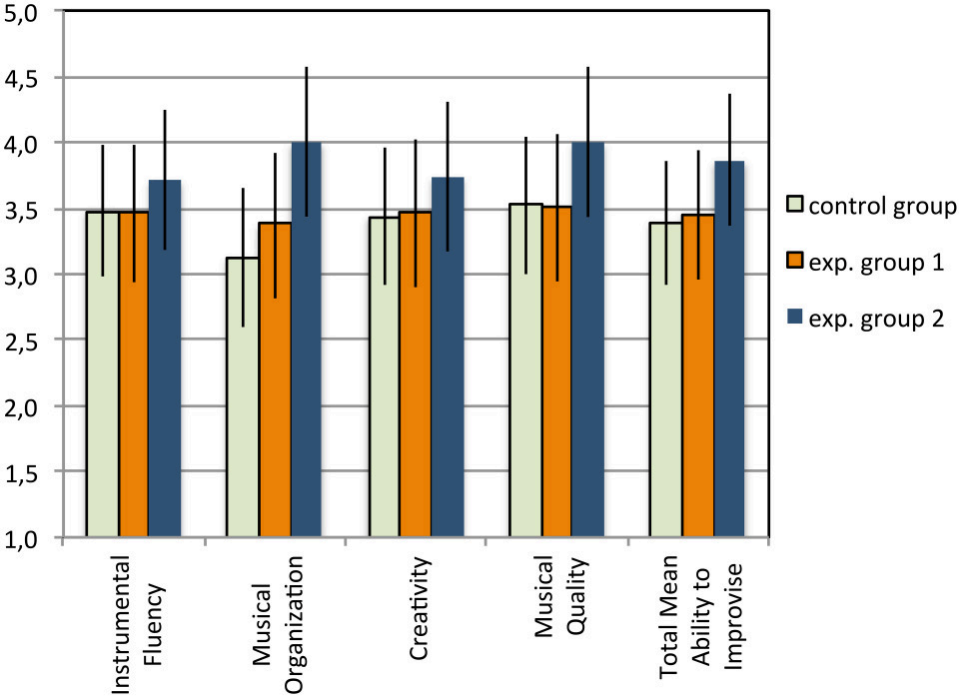
**Solo task: Scores for each evaluative criteria and the Total score of Ability to Improvise (means)**. The error bars indicate 95% confidence intervals.

**Figure 6:**
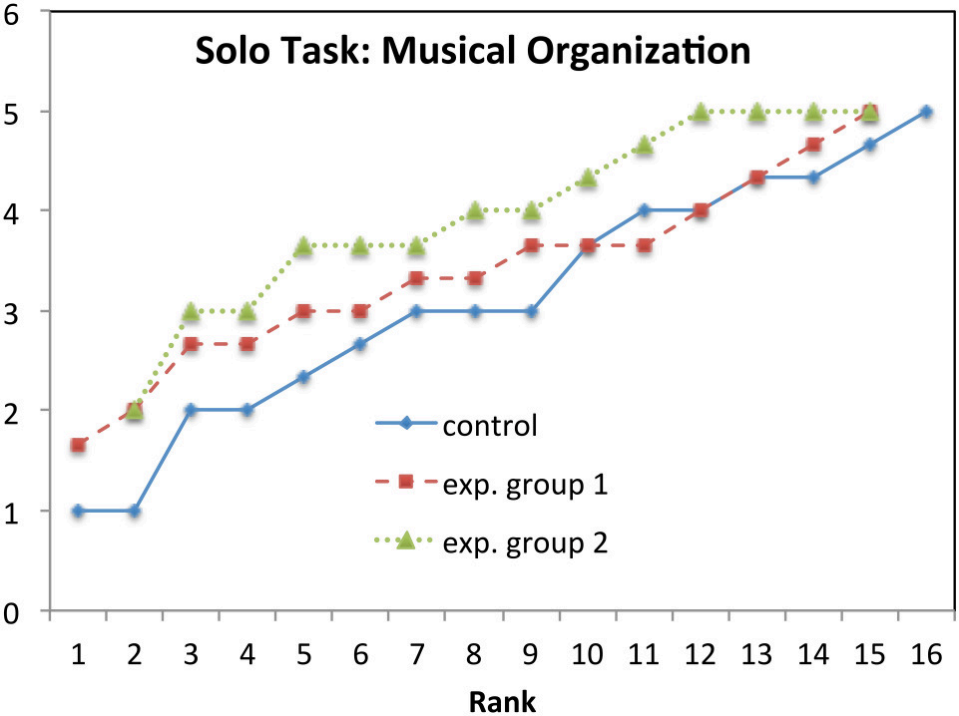
**Solo task, *Musical Organization*: Mean values rank-ordered in each group**.

**Figure 7:**
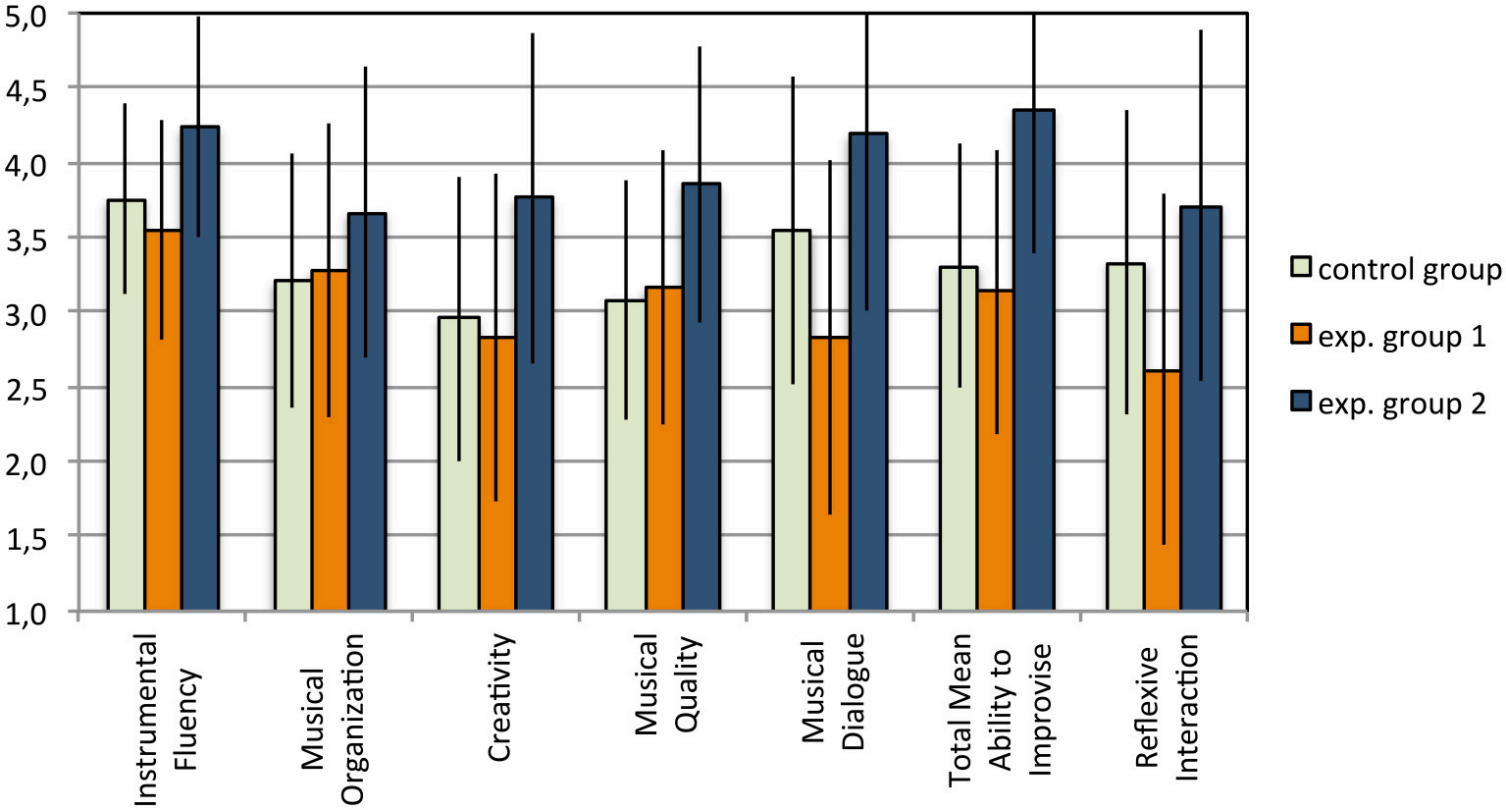
**Duet task: Scores for each evaluative criteria, Total score of Ability to Improvise, and score of Reflexive Interaction (means)**. The error bars indicate the 95% confidence intervals.

**Figure 8:**
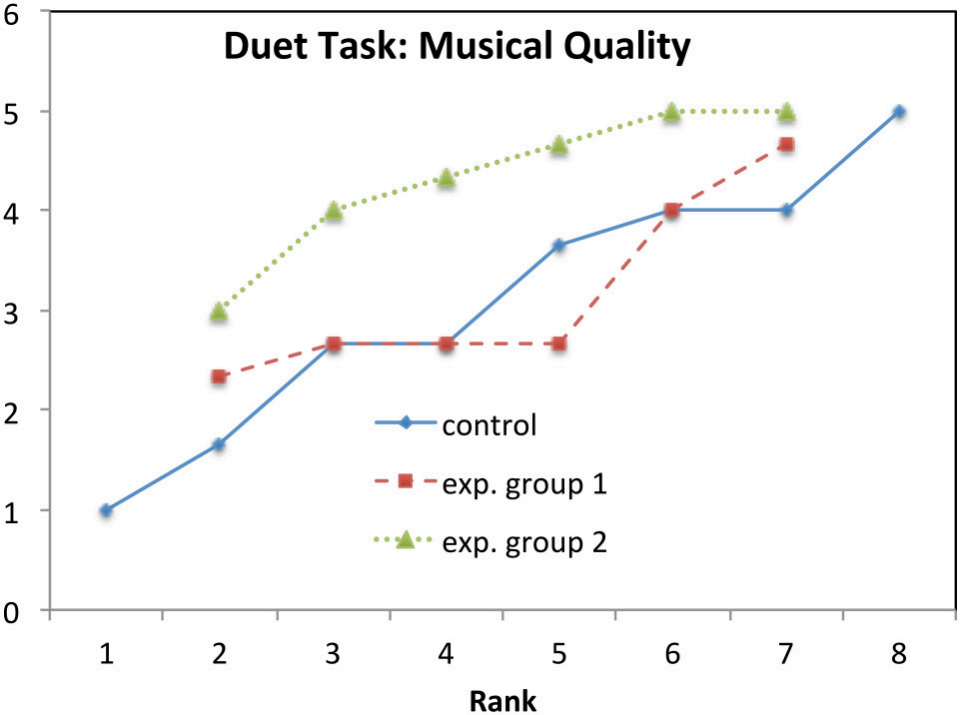
**Duet task: Mean values rank-ordered in each group for Musical Quality**.

**Figure 9:**
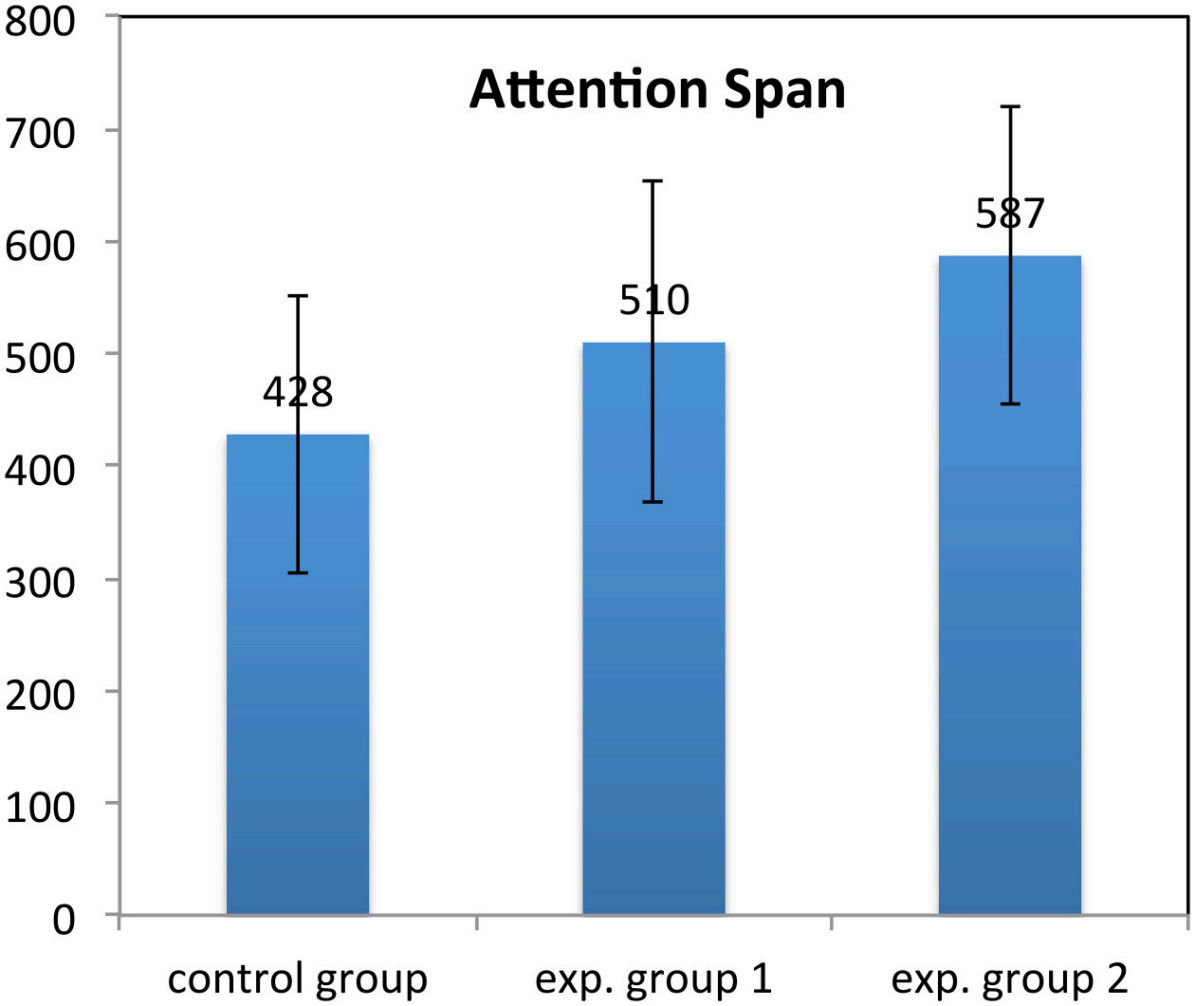
**Duet task: Plot of the attention span in each group**. The columns indicate the duration of children's attention in s. The error bars indicate the 95% confidence intervals.

The authors apologize for these errors and state that these do not change the scientific conclusions of the article in any way.

## Conflict of interest statement

The authors declare that the research was conducted in the absence of any commercial or financial relationships that could be construed as a potential conflict of interest.

